# LED light promoted synthesis of silver nanoparticle with red cabbage extract in clinical conditions and its dental applications

**DOI:** 10.1007/s10266-025-01086-5

**Published:** 2025-03-19

**Authors:** Zeynep Aslı Güçlü, Nur Sultan Gundes, Nimet Temur, Ismail Ocsoy, Nilay Ildız

**Affiliations:** 1 Department of Pediatric Dentistry, Faculty of Dentistry, Erciyes University, 38039 Melikgazi, Kayseri Türkiye; 2 Department of Analytical Chemistry, Faculty of Pharmacy, Erciyes University, Kayseri, Türkiye; 3 Medical Imaging Department, Vocational School of Health Services, Bandırma Onyedi Eylul University, 10200 Bandirma, Türkiye

**Keywords:** Anthocyanins, Antimicrobial property, Chlorhexidine, LED light device, Silver nanoparticle

## Abstract

Silver nanoparticles (Ag NPs) are effective universal germicides toward various pathogens. Herein, we developed synthesis of fast and stable Ag NPs with red cabbage extract (RCE) used as reducing and capping agent promoted by the LED light device used in clinics for dental polymerization and investigated their antimicrobial properties for dentistry purposes. We systematically explained the formation mechanism of anthocyanins (anth) directed, existing as main and predominant components in RCE, Ag NPs (anth@Ag NP) in 10 s (sn) under photoirradiation by LED light with a standard power mode (1000 mW/cm^2^). We tested anth@Ag NP as an effective cavity disinfectant and caries arresting agent with its enhanced antimicrobial property against model cariogenic pathogens including standard strains of *Streptococcus mutans* (*S. mutans*) ATCC 25275, *Enterococcus faecalis* (*E. faecalis*) ATCC 29212, *Staphylococcus aureus* (*S. aureus*) ATCC 29213, and *Candida albicans* (*C. albicans*) ATCC 90028. We claim that the anth@Ag NP can be doped into several dental materials polymerized by LED light for long-term antimicrobial properties toward dental infections.

## Introduction

A contemporary technique to treating deep dentin caries involves a minimally invasive approach that selectively removes the caries, as opposed to the traditional way of totally removing the decay, which carries a risk of exposing the pulp. Depending on the depth and activity of the decay, soft/ infected dentin can be removed entirely, or a certain amount of soft/infected dentin can be left over the pulp to preserve pulp health [1, 2]. It is assumed that a hermetic restoration, placed after the selective cleaning of caries, will stop the progression of caries by isolating the remaining microorganisms in the selectively left carious tissue from the oral environment and reducing their metabolic activity by cutting off their food sources [3–5].

However, the survival ability of microorganisms under restoration is uncertain. Studies indicate that the microorganisms can survive by utilizing glycoproteins and amino acids derived from pulp fluid as nutrients [6–8]. The ability to survive under restoration may be specific to certain species within the microbiota. Additionally, the total microbial load before the restoration is placed, and the sealing between the restoration tooth surface and the overall quality of the restoration affects microbial viability [8]. It is believed that antimicrobial strategies that can enable the disinfection of the lesion by selectively leaving caries while reducing the total microbial load will enhance the effectiveness of minimally invasive treatment options, thereby providing the possibility of halting the progression of caries [9].

The increasing microbial resistance to current antimicrobial agents (antibiotics, metal ions, etc.) has heightened interest in using nanoparticles (NPs) as antimicrobial agents [10]. Silver (Ag) NPs with their intrinsic antimicrobial activities among others have been extensively used for a variety of pathogens [11–15]. Although various methods (physical, chemical, and biological) have been developed for synthesis of Ag NPs [16–20], in general, biological methods have been preferred by the researchers, working on bioanalytical and biomedical applications, for synthesis of biogenic Ag NPs due to non-use of toxic solvent, reducing and stabilizing agents.

Up to now, Ag NPs with various forms have been heavily utilized in dentistry for either biofilm prevention/pathogen destruction or dental materials components [21–28]. To the best of our knowledge, our work is first endeavor to carry out in situ synthesis of stable and uniform anth@Ag NPs via photoreduction process by LED light in 10 sn. The antimicrobial activities of the anth@Ag NP against common cariogenic infections, including *Streptococcus mutans (S. mutans), Enterococcus faecalis (E. faecalis), Staphylococcus aureus (S. aureus), and Candida albicans (C. albicans),* were tested and compared with a 2% chlorhexidine gluconate (CHX), one of the most commonly used cavity disinfectant and caries arresting agent.

## Materials and methods

### Materials and instrumentation

All chemicals were used as received without purification. Silver Nitrate ACS reagent, ≥ 98% (AgNO_3_) used as a precursor for synthesis of anth@Ag NPs was obtained from Sigma-Aldrich, USA. Chlorhexidine (CHX) were obtained from Microvem (Altun Sterilization and Medikal, Turkiye). Red cabbage was purchased from province Kayseri of Türkiye. The VALO LED light device with the serial number C18804 employed for photoreduction in Erciyes University, Faculty of Dentistry was purchased from Ultradent Products, USA. The absorbance of anth@Ag NPs were recorded with UV–Vis spectrophotometer (UV1900i; Shimadzu, Japan). Morphology and elemental analysis of anth@Ag NPs were monitored by scanning transmission electron microscopy (STEM) (Zeiss, Gemini 500) and energy-dispersive X-ray spectroscopy (EDX) (Zeiss, Gemini 500), respectively. Fourier-transform infrared spectroscopy (Perkin Elmer, 400 FTIRSpectrometer Spotlight 400 Imaging System) was used to analyze presence of plant extracts on the surface of the anth@Ag NPs. Hydrodynamic size and surface charge of anth@Ag NPs were measured by dynamic light scattering (DLS) (Malvern, Nano ZS90) and Zeta Potential (Malvern, Nano ZS90), respectively. *S. mutans, E. faecalis, S. aureus, and C. albicans* stored in skim milk medium (Difco^™^) at − 20 ºC obtained from Erciyes University, Faculty of Pharmacy and Pharmaceutical Microbiology research laboratory culture collection.

### Red cabbage extract (RCE) preparation

The red cabbage extract (RCE) was prepared based on previous literature [29–31]. Red cabbage obtained from the local market was washed and cut into small pieces. 100 g of red cabbage and 100 mL of distilled water in glass baker were boiled for 30 min (min). The extract was filtered through (Millex^®^-HV Filter Unit, Sigma-Aldrich, USA). The final extract was stored at − 20 °C for use in Ag NP synthesis. The concentration of the RCE was adjusted by the addition of distilled water.

### Green synthesis of anth@Ag NPs via photoreduction

For the synthesis of anth@Ag NPs, 995 µl of 0.2 mM AgNO_3_ solution and 5 µl of 0.5% (w/w) RCE solutions were mixed into a 2 ml glass vial and it was vortexed (Sigma-Aldrich, USA). The mixture was exposed to an LED light device (VALO LED light device from Ultradent Products, USA) with wavelength range 385–515 nm and standard power mode 1000 mW/cm^2^ for 10 sn. After LED light exposure, the color of the mixture was turned to light-brown, which can be indication of anth@Ag NPs formation. The anth@Ag NPs solution was centrifuged at 14,000 rpm for 10 min to collect anth@Ag NPs as precipitate at the bottom of the tube and to remove excess of Ag + and RCE in supernatant. The anth@Ag NPs were dissolved in distilled water and stored in a refrigerator at + 4 °C for further use.

### Characterization analyses of synthesized anth@Ag NPs

STEM, UV–Vis Spectrophotometer, EDX, FT-IR, DLS, and Zeta Potential were used for characterization of the anth@Ag NPs. Size and shape of anth@Ag NPs were monitored with STEM and elemental analysis was characterized by EDX coupled to STEM. UV–Vis Spectrophotometer was used for absorbance peak of the anth@Ag NPs. The stretching and vibration of anthocyanins bound on surface of the Ag NP was analyzed by FTIR. The effective diameter and surface charge of anth@Ag NPs were determined DLS and Zeta Potential, respectively.

The ant@AgNP sample, which was centrifuged and stored in the refrigerator at + 4 °C, was dried and weighed. After weighing, three different ant@AgNP concentrations of 100 ppm (AgNP1), 75 ppm (AgNP2), and 50 ppm (AgNP3) were prepared by dissolving the dry sample with distilled water.

### Analysis of antimicrobial activity of anth@Ag NPs

Antimicrobial tests were conducted using standard bacterial and fungal strains from the Pharmaceutical Microbiology Laboratory's culture collection at Erciyes University, Mustafa Kılıçer Faculty of Pharmacy. Antimicrobial activities of anth@Ag NPs were determined against standard oral pathogen strains by determining the Minimum Inhibitory Concentration (MIC) and Minimum Bactericidal/Fungicidal Concentration (MBK/MFK) by broth microdilution method according to the Clinical Laboratory Standards Institute (CLSI) guidelines (CLSI M27-A3 [32]; CLSI M07-A9 [33]).

MIC experiment was performed in 96-well U-bottom microplates (Isolab, Germany). The anth@Ag NPs were prepared by diluting the sample stored in the refrigerator at + 4 °C with sterile distilled water in a 1:1 ratio twice. The anth@Ag NPs concentrations used in antimicrobial assays were calculated spectrophotometrically using the Lambert–Beer Law [34].

For comparison of antimicrobial activity, 0.5% w/w RCE solution and 2% CHX solution were used. Cationic Mueller–Hinton broth (CMHB) (Condalab, Spain) was used for testing bacterial strains, while RPMI 1640 broth without sodium bicarbonate and with 0.2% glucose and l-glutamine (Cegrogen Biotech, Germany) was used for *C. albicans* ATCC 90028 strain. All experiments were performed triplicate. The same experimental design was used for all microorganisms. The microplates in which *E. faecalis* ATCC 29212 and *S. aureus* ATCC 29213 strains were tested were incubated at 37 °C in an aerobic environment for 24 h (hrs). The microplate in which *S. mutans* ATCC 25275 strain was tested was incubated at 37 °C in an aerobic environment supported by 5% CO_2_ for 24 h. The *C. albicans* ATCC 90028 strain was incubated at 37 °C in an aerobic environment for 48 h.

At the end of the incubation period, the lids of the microplates were opened and 20 µl of the mixture was taken from all wells for each microorganism using sterile micropipettes. While bacteria were spread on Mueller–Hinton Agar (Condalab, Spain), *C. albicans* was spread on Sabouraud Dextrose Agar (brand). At the end of the incubation period, the anth@Ag NPs concentration in which no microbial growth was detected in the agar medium was evaluated as MBC/MFC. In addition, colony counts were made according to negative and positive controls in Petri dishes and % inhibition values were calculated.

For the determination of the minimum inhibitory concentration (MIC), 40 µl of MTT (0, 2 mg/ml) was added to all wells tested in the microplates. Microplates were left at room temperature (RT: 25 °C) for 10–15 min. At the end of this period, a color change in the mixtures in the test tubes indicated bacterial growth, while remaining colorless was interpreted as the absence of bacterial growth. The MIC value was determined as the last well where no color change was observed.

### Statistical analysis

Graph Pad Prism (version 8.0.1) software was used for the statistical analysis and graphing of antimicrobial analysis data. Experiments were conducted with at least three repetitions, and the One-Way ANOVA test (Tukey's multiple comparison test) was used to evaluate the differences between groups. The statistical significance of the results was assessed at the *p* < 0.05.

## Results

### Synthesis and characterization of nanoparticles

**Fig. 1:**
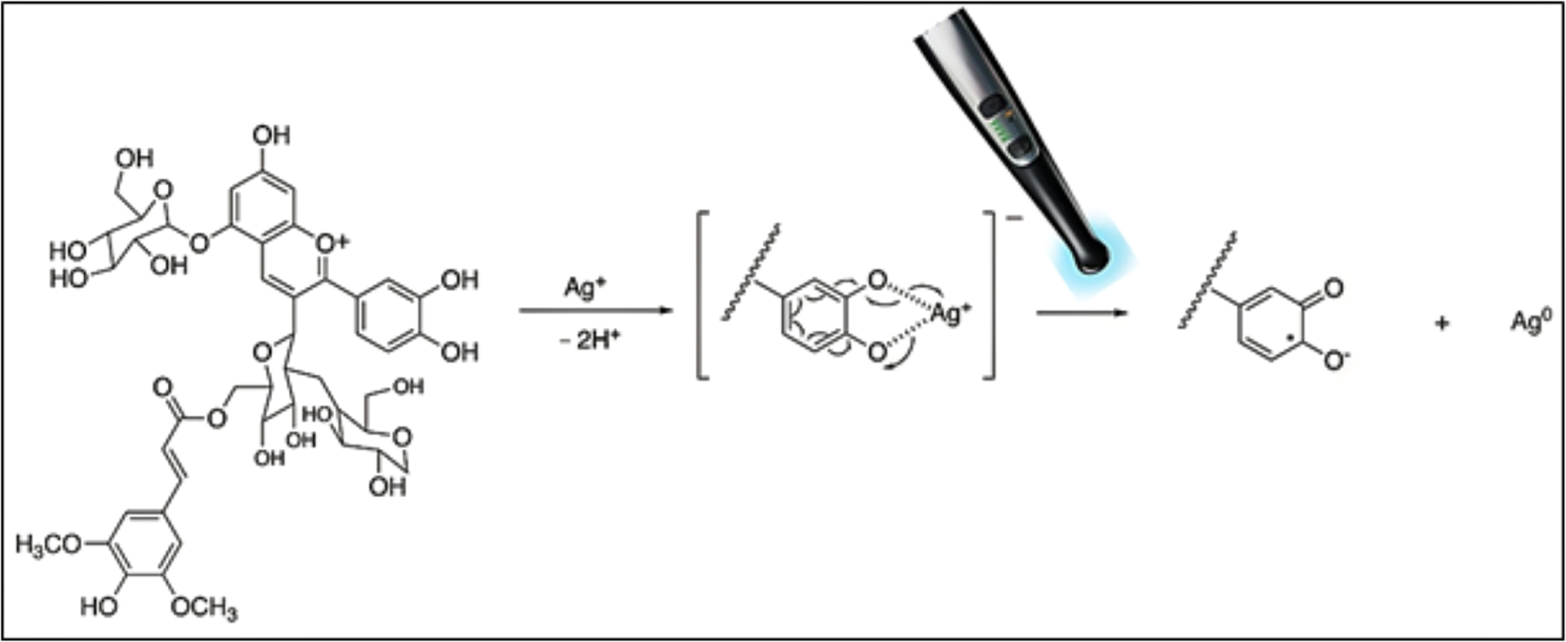
The proposed reaction mechanism for formation of anthocyanin–Ag^+^ complexes and anth@Ag NPs under LED light

In this study, we rapidly synthesized quite stabile and uniform anth@Ag NPs under the photoreduction process by LED light device used in dentistry clinics. Briefly, the catecholamine group of anthocyanin reacts with Ag^+^ ions to form anthocyanin–Ag^+^ complexes, and then, anthocyanin molecule acts as a light-active reductant under irradiation of the LED light device for fast reduction of Ag^+^ to zero valent Ag^0^. The proposed reaction mechanism for formation of anthocyanin–Ag^+^ complexes and eventual anth@Ag NPs is demonstrated in Fig. 1.

**Fig. 2:**
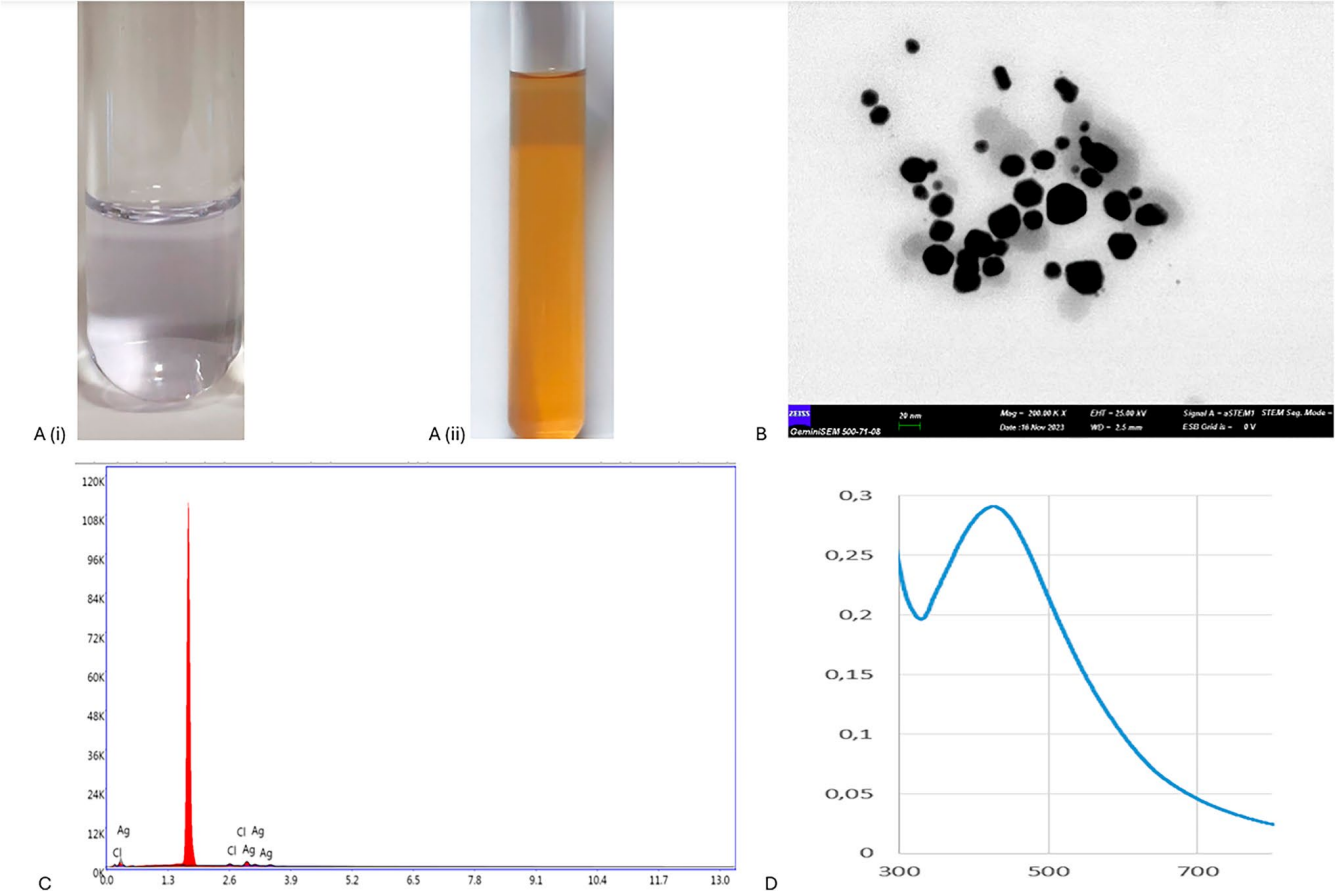
**a** (i). Color of mixture of anthocyanin–Ag^+^ complexes solution and **a** (ii). anth@Ag NP solution, **b** STEM image of anth@Ag NPs, **c** Elemental analysis of Ag metal by EDX in anth@Ag NP, **d** LSPR peak (415 nm) of anth@Ag NPs

The anthocyanin solution was added into Ag^+^ ion solution and the resulting mixture was vortexed for 2 min to both accelerate and facilitate the formation of anthocyanin–Ag^+^ complexes. The light-purple-colored anthocyanin–Ag^+^ complex mixture given in Fig. 2A (i) was exposed to LED light device for 10 sn, and then, the mixture was turned to light-brown given in Fig. 2A (ii), which can be indication of anth@Ag NP formation. STEM image revealed that anth@Ag NPs have quite spherical and a uniform size distribution with diameter of between 15 and 17 nm (Fig. 2B). EDX analysis gave the almost 93% weight percentage of Ag metal in anth@Ag NPs in terms of elemental analysis (Fig. 2C). The spherical Ag NPs give characteristic absorbance peaks in between 400 and 460 nm due to their intrinsic surface plasmon properties. In addition to that, absorbance measurement is commonly used to confirm formation of the Ag NPs prior to STEM operation. The LED light-induced anth@Ag NPs gave narrow and sharp absorbance peak at around 415 nm, as shown in Fig. 2D. The shape profile of absorbance spectrum is well consistent with size distribution or dispersity of the NPs. When the absorbance spectra have narrow and broad shape, the NPs are monodisperse or poly-dispersed, respectively.

**Fig. 3:**
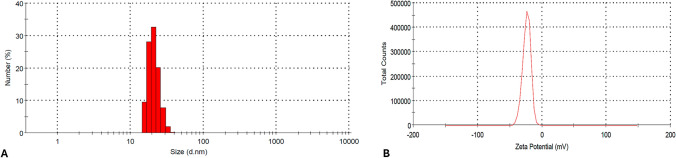
**a** DLS spectrum of anth@Ag NPs and **b** ZT value of anth@Ag NPs

Dynamic size scattering (DLS) measurements provide hydrodynamic size of the NPs in solution. The meaning is that the while solid state size of the NPs was measured on copper grid or steel stub by an electron microscopy like STEM, DLS measures size of the NPs by considering both position and length of ligands bound to surface of the NPs. This is the reason that the DLS size of NPs is always bigger than the solid state size given by electron microscopy. We found that while size of anth@Ag NPs on STEM image was between 15 and 17 nm shown in Fig. 2B, their hydrodynamic size was determined on DLS spectrum to be mostly 25–30 nm (Fig. 3A). The narrow size distribution on DLS spectrum represents monodispersed and colloidal properties of anth@Ag NPs. In addition to that, anth@Ag NPs was also characterized with zeta potential (ZT). The ZT measures electrostatic potential reasoned by charges (positive or negative) around the colloidal particles, conductivity, and viscosity of the particle solution [35]. The anth@Ag NPs have negative charge with a –23.5 mV owing to the presence of deprotonated hydroxyl groups of anthocyanin molecule on Ag NPs (Fig. 3B).

**Fig. 4:**
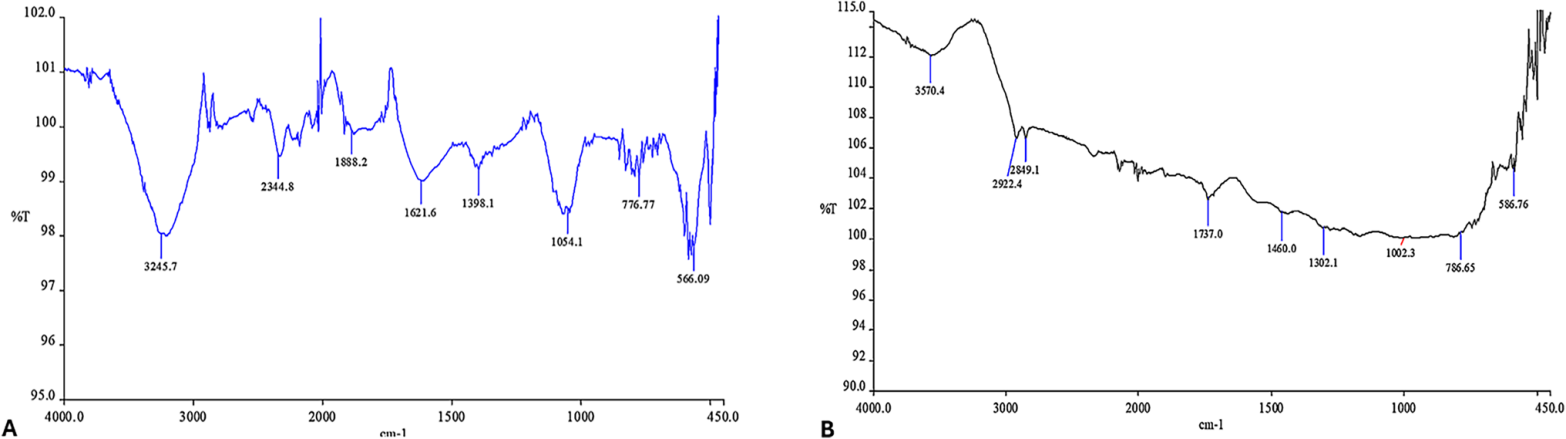
FTIR analysis of A RCE and B anth@Ag NPs

FTIR spectra of RCE and anth@Ag NPs are given in Fig. 4A, B, respectively. In terms of FTIR spectrum of RCE (Fig. 4A), the spectral band seen at 3245.7 cm^−1^ corresponds with O–H bonds. The bands at 2344.8 cm^−1^ and 1888.2 cm^−1^ show the carbonyl groups (C = O). It might come from the ketone groups attributed to phenolic chemicals in RCE. The stretching of C = C double-bond peak was seen at 1621.6 cm^−1^. The stretching vibrations of the C = N double-bond peak was recorded at the 1398.1 cm^−1^. Bonds between C–O show bending peak at 1054.1 cm^−1^.

For FTIR analysis of anth@Ag NPs (Fig. 4B), O–H of water molecules on the surface of Ag NPs gave a peak at 3570.4 cm^−1^. The vibrational peaks of C–H bonds are seen at 2849.1, 2922.4, 1460.0, and 1002.3 cm^−1^. The presence of a band at 1737.0 cm^−1^ suggests the existence of a carbonyl C = O group. The stretching vibrations seen at a wavenumber of 1302.1 cm^−1^ typically suggest the presence of C–N bond. The vibration at 786.65 cm^−1^ generally indicates metal–nitrogen (M–N) bonds. It can demonstrate the presence of nitrogen-containing compounds on the surface of Ag NPs. The 586.76 cm^−1^ vibrational mode indicates the oxidation state of Ag NPs or the presence of metal oxides.

### Antimicrobial activity of anth@Ag NPs

**Fig. 5:**
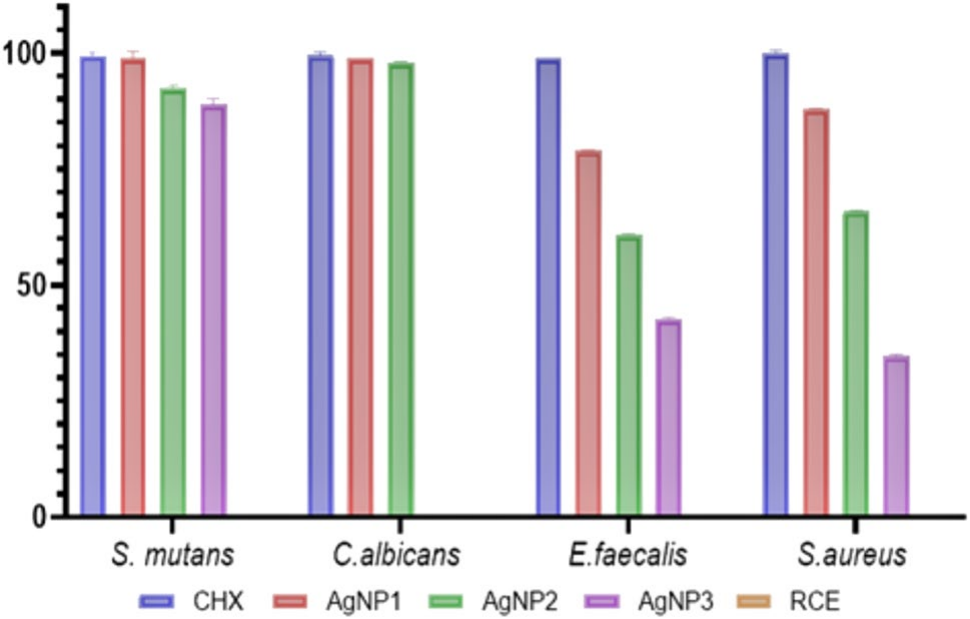
Statistical analysis results. In the diagram, the RCE expressed in yellow indicates a zero level of antimicrobial activity, as it did not demonstrate any efficacy

Percentage inhibition values determined by counting colonies on petri dishes based on negative and positive controls are shown in Table 1 and Fig. 5. In addition, statistical analysis results are shown in Table 2. On all tested microorganisms, 0.5% (w/w) RCE used in the synthesis did not show antimicrobial activity. Table 1 and Fig. 5 reveals that Ag NP1 (100 ppm) and Ag NP2 (75 ppm) effectively inhibited almost 99% and 97% of *C. albicans*, respectively. Additionally, Ag NP1 and Ag NP2 caused 99% and 93%, 88% and 66%, and 79% and 61% of cell death for *S. mutans, S. aureus, and E. faecalis,* respectively. While Ag NP3 (50 ppm) did not show antimicrobial activity on *C. albicans,* it inhibited 89%, 43% and 35% of bacterial cells of *S. mutans, S. aureus, and E. faecalis*, respectively.

**Table 1:** Percentage inhibition values of microorganisms

	CHX	Ag NP1	Ag NP2	Ag NP3	RCE
*S. mutans*	99,445 ± 0,7	98,995 ± 1,4	92,46 ± 0,65	88,94 ± 1,32	0
*C. albica*ns	99,55 ± 0,77	98,995 ± 0,007	97,99 ± 0,14	0	0
*E. faecalis*	98,99 ± 0,014	78,89 ± 0,15	60,8 ± 0,3	42,71 ± 0,41	0
*S. aureus*	99,98 ± 0,7	87,935 ± 0,09	65,825 ± 0,24	34,67 ± 0,46	0

**Table 2:** Statistical analysis results

Tukey’s multiple comparisons’ test	Mean diff	95.00% CI of diff	Summary	P value
CHX vs. Ag NP1	8,287	−32,95–49,53	ns	0,9695
CHX vs. Ag NP2	20,22	−21,02–61,46	ns	0,5695
CHX vs. Ag NP3	57,91	16,67–99,15	**	**0,0045**
CHX vs. RCE	99,49	58,25–140,7	****	** < 0,0001**
Ag NP1 vs. Ag NP2	11,94	−29,31–53,18	ns	0,8949
Ag NP1 vs. Ag NP3	49,62	8,382–90,87	*	**0,0151**
Ag NP1 vs. RCE	91,20	49,96–132,4	****	** < 0,0001**
Ag NP2 vs. Ag NP3	37,69	−3,553–78,93	ns	0,0814
Ag NP2 vs. RCE	79,27	38,03–120,5	***	**0,0002**
Ag NP3 vs. RCE	41,58	0,3379–82,82	*	**0,0477**

*ns* no significant

Colony counts and percentage of inhibition values ( the significance is shown in bold, and the asterisk shows the degree of efficiency)

Figure 5C presents that no significant difference was found between Chlorhexidine (CHX) and Ag NP1 (100 ppm) and CHX and Ag NP2 (75 ppm) in terms of antimicrobial activity on all tested microorganisms. Ag NP1 and Ag NP2 showed similar antimicrobial activity to CHX. It was observed to have CHX significantly higher antimicrobial activity than Ag NP3 on the microorganisms on which Ag NP3 showed activity (*p* < 0.045). On all tested microorganisms, no significant difference was found between Ag NP1 and Ag NP2 in terms of antimicrobial activity.

Ag NP1 was found to be significantly more effective than Ag NP3 on all tested microorganisms (*p* < 0.0151). Ag NP3 did not show antimicrobial activity on C. albicans. No significant difference was found between Ag NP2 and Ag NP3 in terms of antimicrobial activity on microorganisms on which Ag NP3 was effective.

## Discussion

The NP synthesis methods mainly utilize the thermal reduction of metal ions [36]. Additionally, chemical, electrochemical, microwave-assisted, and photochemical reduction methods have also been used [36, 37]. The photoreduction can be considered as a simple, rapid, cost-effective method [37, 38]. The method supports green synthesis as no chemical substances are used, and no toxic products or by-products are formed during the NP synthesis.

In the management of caries, laboratory and clinical studies related to silver compounds have been examined, and it has been observed that substances containing silver particles, such as AgNO_3_ (Silver Nitrate), AgF (Silver Fluoride), and SDF (Silver Diamine Fluoride), have been researched and utilized. The most notable disadvantage of silver compounds is that they cause black discoloration on decayed tissue. This coloration is due to the oxidation of ionic silver to metallic silver [39]. SDF, a silver compound, is an easy-to-apply, cost-effective, minimally invasive treatment option to stop the progression of cavities in deciduous and permanent teeth. Although SDF is promising in managing caries in young children, but it contains silver in a high concentration (38%), which causes discoloration in the teeth where it is applied [40]. Schwass et al., [9] compared the amount of Ag^+^ ions released from Ag NP formulation, developed to disinfect carious dentin, with 38% of silver in SDF. The work suggests that Ag NPs (38.4 μg/ml) release Ag^+^ ions at a much lower concentration than SDF (320,000 μg/ml) and this amount may not cause dark staining. Santos et al. [41] reported a randomized clinical study using the nano-silver-fluoride formulation to effectively arrest dentin caries. They also noted that this formulation does not form oxidized product when it comes into contact with oxygen in the environment and may not cause dark staining on dental tissues.

It is well known that various plant extracts containing bioactive metabolites, such as carboxylic acid, ascorbic acid, ketones, alkaloids, aldehydes, and flavonoids (phenolic compounds), have been used for synthesis of biogenic Ag NPs [42, 43]. We preferred RCE as a reducing and stabilizing agent in the synthesis of the Ag NPs owing to its anthocyanins content with core of cyanidin3-O-diglucoside-5-O-glucoside. The anthocyanin molecule easily reacts with different metal ions to form the anthocyanin–metal complexes due to high hydroxyl groups on its structure and leads the formation of NPs [30, 44–46].

It has been reported that various light sources, such as sunlight, traditional fluorescent light, and UV light, induce the photoreduction of Ag^+^ ions and leading the formation of Ag NPs [47–50]. The best of our knowledge, we study, for the first time, in situ and a single step synthesis of Ag NP from RCE in a very short time under the photoreduction process using VALO LED (Ultradent Product, USA) light device used for polymerization of dental resin materials in dental clinics [45, 51].

In our study, anth@Ag NPs synthesized by LED light photoreduction showed similar antimicrobial activity to CHX at concentrations of AgNP1 and AgNP2 on all tested microorganisms. Due to studies showing that chlorhexidine (CHX) can affect the sealing of adhesive systems, this result is promising as an alternative to CHX for caries disinfection, with further in vitro and in vivo studies supporting this result. There are studies in the literature comparing the antimicrobial efficacy of Ag NPs and CHX. Panpaliya and his colleagues [52] evaluated the antimicrobial efficacy of AgNPs (30–50 nm in size) against five different oral pathogenic bacteria (*S. mutans, S. oralis, L. acidophilus, L. fermentum, and C. albicans*) in their in vitro study, comparing it to CHX (2%). Ag NPs have shown better antimicrobial effects at five times lower concentrations than CHX.

Souza and colleagues [53] synthesized Calcium Glycerophosphate-AgNP nanocomposites (approximately 50 nm in size, spherical) using extracts obtained from the leaves, bark, and seeds of the pomegranate plant through a green method. The study investigated these compounds' antimicrobial/antibiofilm activity against *S. mutans* and *C. albicans*. These nanostructures have been effective against *S. mutans* biofilms with results similar to or better than CHX. However, it has not shown effectiveness against biofilms formed by *C. albicans*. Additionally, the results of the study have shown a dose-dependent inhibitory effect. Rodrigues and colleagues [54] evaluated the antimicrobial effects of an AgNP-containing irrigation agent, sodium hypochlorite, and CHX against the biofilm of *E. faecalis* and its planktonic form in infected dentin tubules. After 5 min, the study found that the AgNP solution inhibited planktonic bacteria in the dentin tubules more efficiently than the biofilm. The AgNP solution demonstrated a lower inhibitory effect on planktonic bacteria than chlorhexidine, but it was more effective in eliminating biofilm (*P* < 0.05).

Anth@AgNPs at concentrations of 100 ppm (AgNP1) and 75 ppm (AgNP2) showed the lowest antimicrobial activity on *E. faecalis* (approximately 79% and 61%). We think that this is related to *E. faecalis* developing resistance mechanisms against harsh conditions, such as alkaline pH [55] and long-term starvation [56].

Laboratory and clinical studies on silver compounds have been examined in the management of caries, such as silver nitrate (AgNO_3_), silver fluoride (AgF), and silver diamine fluoride (SDF). The most apparent disadvantage of silver compounds is dark coloration of the dentine tissue. This coloration results from the oxidation of ionic silver to metallic silver [57]. GDF, one of the silver compounds, is an easy, cost-effective, minimally invasive treatment option to arrest the progression of caries in primary and permanent teeth. Due to these advantages, it is promising in managing caries in young pediatric patients. However, it contains a high concentration of Ag^+^ (38%) and causes discoloration on the teeth when applied [58]. Schwass et al. [59] developed an AgNP formulation to disinfect dentin. When they compared the release of Ag + ions with SDF, they found that the AgNP formulation releases a significantly lower concentration of Ag + than SDF. They suggested that this level of ion release does not lead to dark coloration. Santos et al. [60] reported that the nano-compound was effective in halting dentin caries in a randomized clinical trial when nano-silver-fluoride was used. They also stated that the nano-compound does not form oxides when it comes into contact with environmental oxygen and may not cause dark discoloration of teeth like SDF. These studies are promising advantages regarding the antimicrobial efficacy provided by nanomaterials.

Our investigation found that the AgNPs produced using LED-assisted photoreduction showed comparable antibacterial effects to CHX against all microbes tested. This was observed at AgNP1 and AgNP2. Nevertheless, when the concentration reached AgNP3, the antibacterial effectiveness declined compared to CHX (*p* = 0.0045).

## Conclusion

We have successfully demonstrated that the LED light device is not only successful for polymerizing dental resins, but also enables the rapid synthesis of anth@Ag NPs in 10 sn using RCE via photoreduction process. This study demonstrates a novel, rapid, and cost-effective method. Anth@Ag NPs, particularly Ag NP1, exhibited significant antibacterial activity against key oral pathogens, comparable to 2% chlorhexidine. This suggests a promising approach for developing new antimicrobial agents for caries arrest and cavity disinfection in clinical settings.

## Data Availability

The datasets used and analyzed during the current study are available from the corresponding author on reasonable request.
